# Supplementary material for: Genomics of Breast Cancer Brain Metastases: A Meta-Analysis and Therapeutic Implications
Source: Cancers (Basel). 2023 Mar 12;15(6):1728. doi: 10.3390/cancers15061728 (PMC10046845; doi:10.3390/cancers15061728)
Supplement: Supplementary file 1 [file cancers-15-01728-s001.zip › cancers-2192941-Figure s1.pdf]

Supplementary Materials:

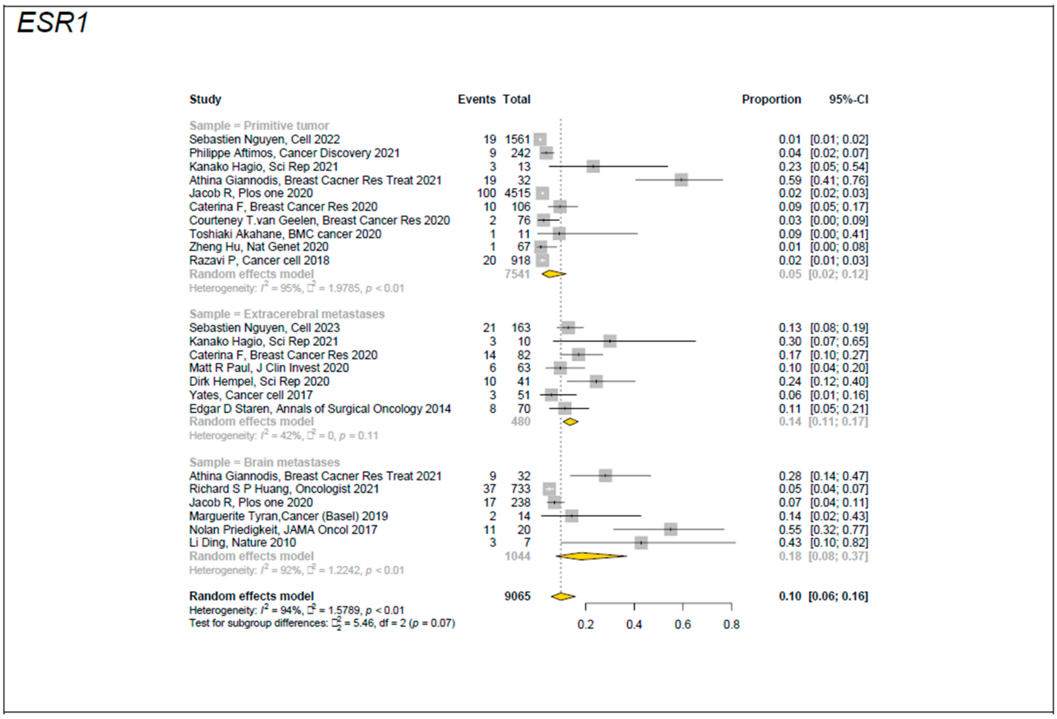

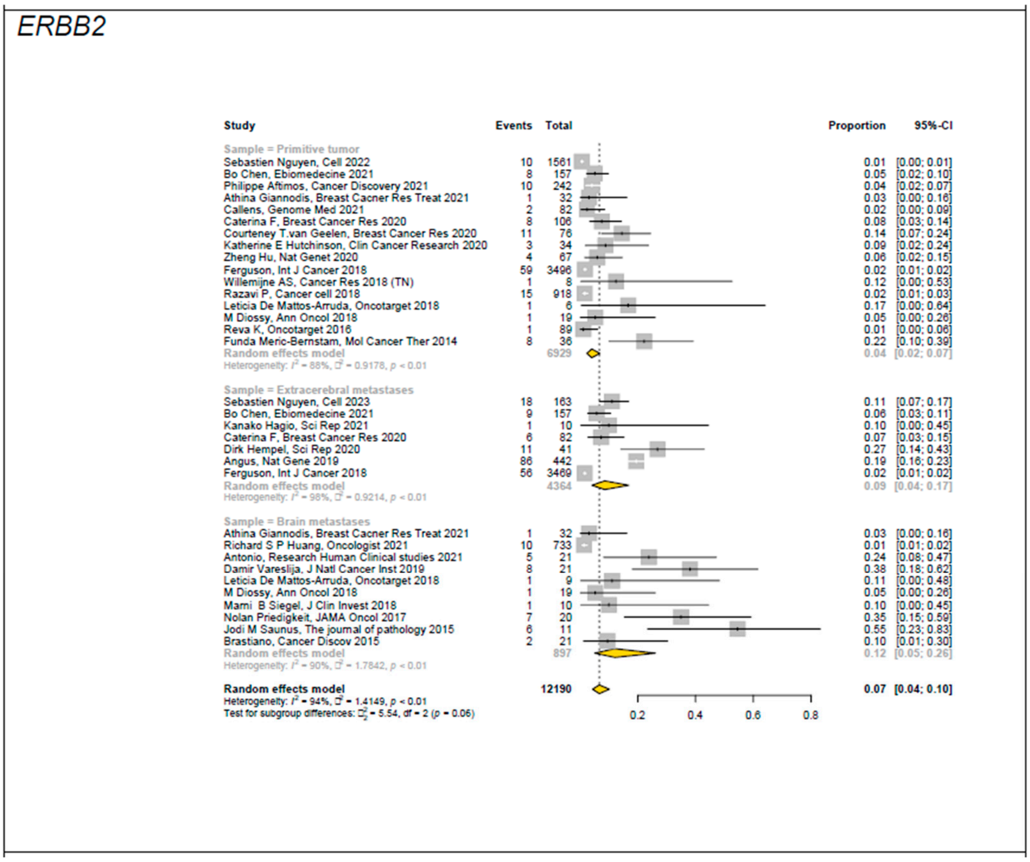

EGFR

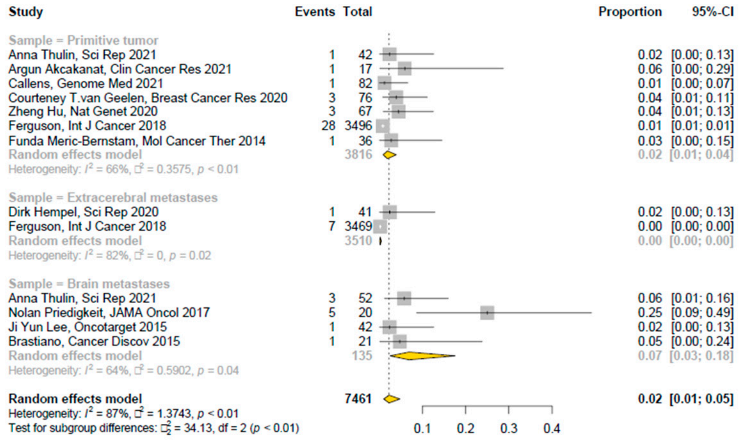
Study Events Total Proportion 95%-CI
Sample = Primitive tumor
Anna Thulin, Sci Rep 2021 1 42 0.02 [0.00; 0.13]
Argun Akcakanat, Clin Cancer Res 2021 1 17 0.06 [0.00; 0.29]
Callens, Genome Med 2021 1 82 0.01 [0.00; 0.07]
Courteney T.van Geelen, Breast Cancer Res 2020 3 76 0.04 [0.01; 0.11]
Zheng Hu, Nat Genet 2020 3 67 0.04 [0.01; 0.13]
Ferguson, Int J Cancer 2018 28 3496 0.01 [0.01; 0.01]
Funda Meric-Bernstam, Mol Cancer Ther 2014 1 36 0.03 [0.00; 0.15]
Random effects model 3816 0.02 [0.01; 0.04]
Heterogeneity: I^2 = 66%, τ^2 = 0.3575, p < 0.01
Sample = Extracerebral metastases
Dirk Hempel, Sci Rep 2020 1 41 0.02 [0.00; 0.13]
Ferguson, Int J Cancer 2018 7 3469 0.00 [0.00; 0.00]
Random effects model 3510 0.00 [0.00; 0.00]
Heterogeneity: I^2 = 82%, τ^2 = 0, p = 0.02
Sample = Brain metastases
Anna Thulin, Sci Rep 2021 3 52 0.06 [0.01; 0.16]
Nolan Priedigkeit, JAMA Oncol 2017 5 20 0.25 [0.09; 0.49]
Ji Yun Lee, Oncotarget 2015 1 42 0.02 [0.00; 0.13]
Brastiano, Cancer Discov 2015 1 21 0.05 [0.00; 0.24]
Random effects model 135 0.07 [0.03; 0.18]
Heterogeneity: I^2 = 64%, τ^2 = 0.5902, p = 0.04
Random effects model 7461 0.02 [0.01; 0.05]
Heterogeneity: I^2 = 87%, τ^2 = 1.3743, p < 0.01
Test for subgroup differences: χ^2_2 = 34.13, df = 2 (p < 0.01)
0.1 0.2 0.3 0.4

PTEN

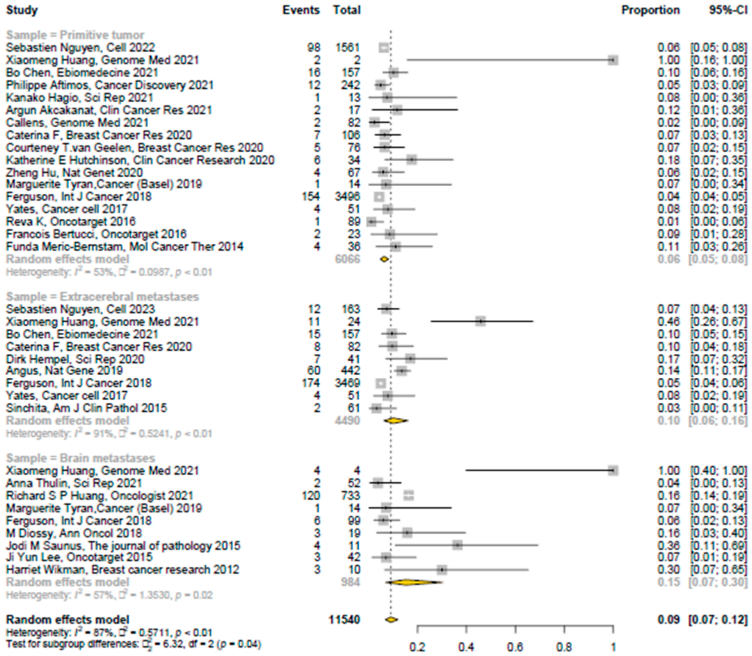
Study Events Total Proportion 95%-CI
Sample = Primitive tumor
Sebastien Nguyen, Cell 2022 98 1561 0.06 [0.05; 0.08]
Xiaomeng Huang, Genome Med 2021 2 2 1.00 [0.16; 1.00]
Bo Chen, Ebiomedecine 2021 16 157 0.10 [0.06; 0.16]
Philippe Aftimos, Cancer Discovery 2021 12 242 0.05 [0.03; 0.09]
Kanako Hagio, Sci Rep 2021 1 13 0.08 [0.00; 0.36]
Argun Akcakanat, Clin Cancer Res 2021 2 17 0.12 [0.01; 0.36]
Callens, Genome Med 2021 2 82 0.02 [0.00; 0.09]
Caterina F, Breast Cancer Res 2020 7 106 0.07 [0.03; 0.13]
Courteney T.van Geelen, Breast Cancer Res 2020 5 76 0.07 [0.02; 0.15]
Katherine E Hutchinson, Clin Cancer Research 2020 6 34 0.18 [0.07; 0.35]
Zheng Hu, Nat Genet 2020 4 67 0.06 [0.02; 0.15]
Marguerite Tyran,Cancer (Basel) 2019 1 14 0.07 [0.00; 0.34]
Ferguson, Int J Cancer 2018 154 3496 0.04 [0.04; 0.05]
Yates, Cancer cell 2017 4 51 0.08 [0.02; 0.19]
Reva K, Oncotarget 2016 1 89 0.01 [0.00; 0.06]
Francois Bertucci, Oncotarget 2016 2 23 0.09 [0.01; 0.28]
Funda Meric-Bernstam, Mol Cancer Ther 2014 4 36 0.11 [0.03; 0.26]
Random effects model 6066 0.06 [0.05; 0.08]
Heterogeneity: I^2 = 53%, τ^2 = 0.0987, p < 0.01
Sample = Extracerebral metastases
Sebastien Nguyen, Cell 2023 12 163 0.07 [0.04; 0.13]
Xiaomeng Huang, Genome Med 2021 11 24 0.46 [0.26; 0.67]
Bo Chen, Ebiomedecine 2021 15 157 0.10 [0.05; 0.15]
Caterina F, Breast Cancer Res 2020 8 82 0.10 [0.04; 0.18]
Dirk Hempel, Sci Rep 2020 7 41 0.17 [0.07; 0.32]
Angus, Nat Gene 2019 60 442 0.14 [0.11; 0.17]
Ferguson, Int J Cancer 2018 174 3469 0.05 [0.04; 0.06]
Yates, Cancer cell 2017 4 51 0.08 [0.02; 0.19]
Sinchita, Am J Clin Pathol 2015 2 61 0.03 [0.00; 0.11]
Random effects model 4490 0.10 [0.06; 0.16]
Heterogeneity: I^2 = 91%, τ^2 = 0.5241, p < 0.01
Sample = Brain metastases
Xiaomeng Huang, Genome Med 2021 4 4 1.00 [0.40; 1.00]
Anna Thulin, Sci Rep 2021 2 52 0.04 [0.00; 0.13]
Richard S P Huang, Oncologist 2021 120 733 0.16 [0.14; 0.19]
Marguerite Tyran,Cancer (Basel) 2019 1 14 0.07 [0.00; 0.34]
Ferguson, Int J Cancer 2018 6 99 0.06 [0.02; 0.13]
M Diossy, Ann Oncol 2018 3 19 0.16 [0.03; 0.40]
Jodi M Saunus, The journal of pathology 2015 4 11 0.36 [0.11; 0.69]
Ji Yun Lee, Oncotarget 2015 3 42 0.07 [0.01; 0.19]
Harriet Wikman, Breast cancer research 2012 3 10 0.30 [0.07; 0.65]
Random effects model 984 0.15 [0.07; 0.30]
Heterogeneity: I^2 = 57%, τ^2 = 1.3530, p = 0.02
Random effects model 11540 0.09 [0.07; 0.12]
Heterogeneity: I^2 = 87%, τ^2 = 0.5711, p < 0.01
Test for subgroup differences: χ^2_2 = 6.32, df = 2 (p = 0.04)
0.2 0.4 0.6 0.8 1

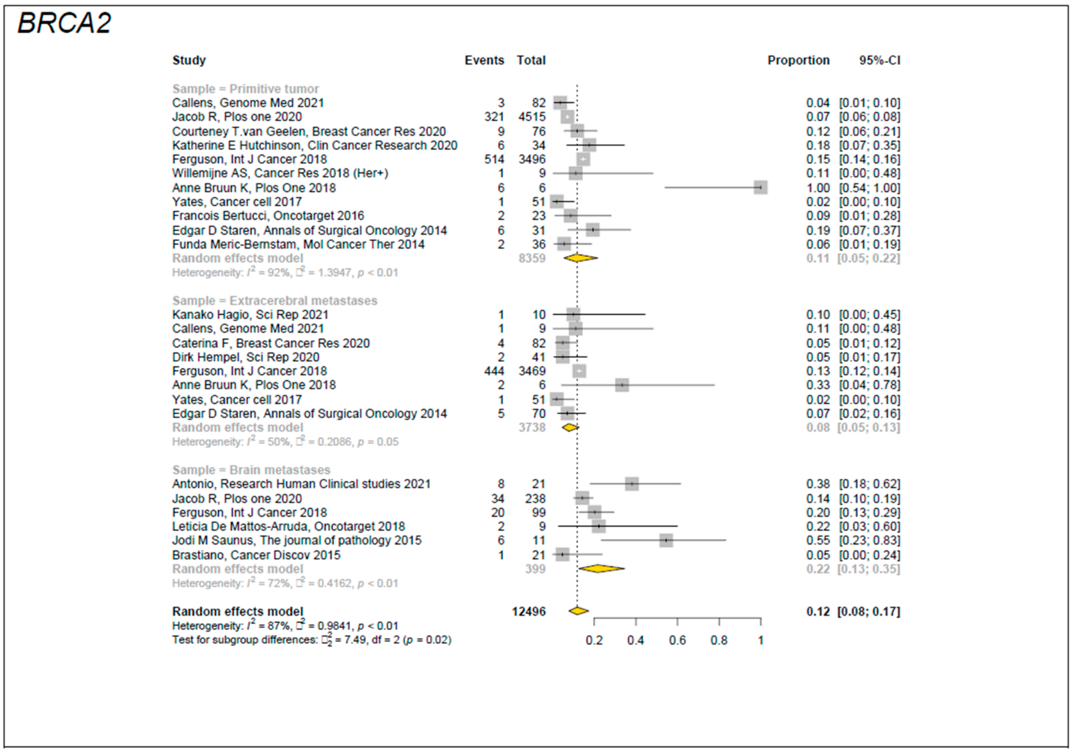

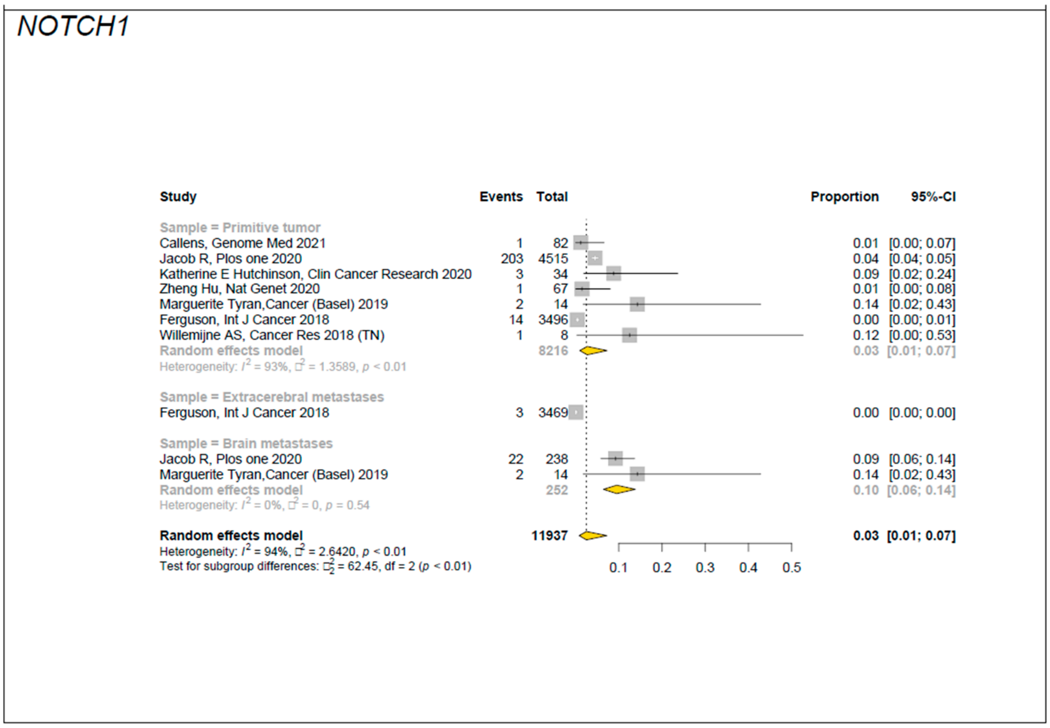

Figure S1. Forest plots of mutation prevalence for 6 genes: *ESR1*, *ERBB2*, *EGFR*, *PTEN*, *BRCA2* and *NOTCH1*.
